# An in vitro analysis of marginal and internal fit of 3D-printed permanent molar endocrowns with different preparation designs

**DOI:** 10.1007/s00784-025-06414-1

**Published:** 2025-05-30

**Authors:** Izim Turker Kader, Safa Ozer, Burcin Arican

**Affiliations:** 1 Department of Prosthodontics, Bahçeşehir University School of Dental Medicine, Istanbul, Turkey; 2 Dental Prosthesis Technology, Bahçeşehir University Vocational School of Health Services, Istanbul, Turkey; 3 Department of Endodontics, Bahçeşehir University School of Dental Medicine, Istanbul, Turkey

**Keywords:** Printing, Three-Dimensional, Prosthodontics, Endocrowns, Marginal gap, Internal gap

## Abstract

**Objectives:**

The purpose of this study was to evaluate the influence of different preparation designs on the marginal and internal fit of 3D-printed permanent endocrowns.

**Materials and methods:**

Typodont maxillary right first molars were prepared and divided into four groups based on different preparation designs: Group 1– butt joint with 2 mm pulp chamber depth, Group 2– butt joint with 4 mm depth, Group 3– shoulder with 2 mm depth, and Group 4– shoulder with 4 mm depth (*n* = 16 each). The prepared teeth were scanned and replicated as 3D-printed resin dies. Permanent endocrowns were fabricated using a ceramic-filled hybrid material and seated with light-body silicone. All restorations were rescanned, and superimposition was performed using 3D analysis software to evaluate marginal, internal, pulp chamber, and overall gaps based on multipoint measurements at standardized locations. Data were statistically analyzed using Two-Way Robust ANOVA (*p* < 0.05).

**Results:**

No significant differences were found between preparation types and depths for internal and overall gap values (*p* > 0.05). However, the highest marginal gap was measured in Group 1 (0.08 mm; *p* = 0.017), while the largest pulp chamber gap was recorded in Group 4 (0.15 mm; *p* < 0.001).

**Conclusions:**

A 1 mm shoulder preparation with a 2 mm pulp chamber depth demonstrated superior marginal and pulp chamber fit compared to other designs. While internal fit did not significantly vary among groups, this design showed the most consistent adaptation overall, supporting its clinical preference for 3D-printed permanent endocrowns.

**Clinical relevance:**

Different preparation designs may affect the fit of endocrowns. Clinicians can prefer an endocrown preparation design of 1 mm shoulder preparation with a 2 mm pulp chamber to improve the adaptation of 3D-printed permanent endocrown restorations.

## Introduction

The common problem in restoring endodontically treated teeth is the higher risk of biochemical deterioration, which might be attributed to the access cavity preparation that increases fracture incidence [1]. Therefore, when considering the restoration of such teeth, restorative materials should be capable of replacing the loss of the tooth structure to ensure mechanical, esthetics, functional properties, and coronal seal [2, 3].

Endocrowns, introduced over two decades ago, are monolithic ceramic restorations designed for the rehabilitation of structurally compromised, endodontically treated teeth [4]. These restorations consist of a coronal portion with an apical extension that fits into the pulp chamber, achieving macromechanical retention. Adhesive cementation further enhances retention by bonding to the wide surface area of the pulp chamber walls, increasing micromechanical interlocking [5, 6]. The depth and configuration of the pulp chamber, as well as the margin design, influence the mechanical performance, marginal and internal adaptation of endocrowns [7–10]. Poor marginal adaptation can precipitate plaque accumulation, thereby increasing the risk of secondary caries, periodontal disease, and endodontic complications, all of which may hinder the long-term success of these restorations [11, 12].

Two commonly studied preparation types are the butt joint, which offers a simplified design with a flat margin, and the shoulder preparation, which provides additional axial wall height and potentially increased surface area for bonding [13, 14]. While some studies suggest that butt joint preparations provide superior marginal fit [13, 15], others have reported improved retention and stress distribution with shoulder designs [10, 14]. However, the comparative performance of these designs in conjunction with emerging three-dimensional (3D)-printed materials has not been fully explored.

Additive manufacturing, particularly 3D printing, has introduced notable advantages in dental restorations by improving accuracy, efficiency, and material use [16, 17]. 3D-printed restorations have demonstrated promising results in terms of marginal and internal fit, largely due to their digital precision and layer-by-layer construction process [16–22]. These benefits may directly affect the clinical performance and longevity of restorations, including endocrowns. Assessing how different preparation designs perform when fabricated through advanced 3D printing techniques may provide new insights into their adaptation potential.

To the best of the authors’ knowledge, while there are studies in the literature comparing the effects of different preparation types on the marginal and internal fit of endocrowns, no study has evaluated 3D-printed ceramic-filled permanent endocrowns using the 3D digital analysis technique. The 3D-printed material used in this study, while not yet supported by studies as a permanent material, is manufacturer-approved for permanent use. Starting from this point, the present study aims to evaluate the internal and marginal fit of 3D-printed permanent molar endocrowns produced over different preparation designs. The null hypotheses for this study were as follows:

1. There would be no difference in the marginal fit of 3D-printed permanent endocrowns between different preparation designs.
2. There would be no difference in the internal fit of 3D-printed permanent endocrowns between different preparation designs.

## Materials and methods

The sample size calculation was performed using a statistical software program (G*Power v3.1.9.2) using data from another study by Oguz et al. [23] the minimum sample size of 16 specimens for each group achieved 95% power to detect differences, with a significance level of 0.05, to test the null hypotheses.

Prior to the present study, a pilot study was conducted with four samples for each group. During this pilot study, one author (S.Ö) gained experience by performing the final preparations after multiple preparation trials under a dental microscope and scanning them with a digital intraoral scanner. Master dies were designed and fabricated as single and in sets of four. An attempt was made to produce all prepared samples as single master dies. However, there were no significant differences between them. In addition, preparing the master dies in sets of four was preferred for each group because it provided ease of measurement. 3D-printed models were produced, and the endocrowns were adhered using light-body impression material. To facilitate easy separation of the endocrowns from the model surface, water, Vaseline, and gel were tested. The best and most controlled results were achieved with Vaseline. Based on these findings, the main study proceeded as outlined below.

The two preparation types—butt joint and shoulder—were selected based on their frequent clinical use and differing biomechanical characteristics. The butt joint provides a flat margin with simplified geometry, whereas the shoulder design introduces additional axial wall height, potentially enhancing bonding surface and retention [13, 14]. These designs were chosen to simulate common clinical scenarios and to allow evaluation of their influence on the fit of 3D-printed endocrowns. The typodont maxillary first molar teeth (AG-3 ZE, Frasaco GmbH, Tettnang, Germany) were prepared according to different preparation designs as Group 1– butt joint with 2 mm pulp chamber depth, Group 2– butt joint with 4 mm pulp depth, Group 3–1 mm shoulder with 2 mm pulp chamber depth, and Group 4–1 mm shoulder with 4 mm pulp chamber depth. All preparations were performed under a dental operation microscope (Zumax OMS 2000, Zumax, China) at 18.4 × magnification by one operator (S.Ö). The preparation for each group commenced with a 2 mm occlusal reduction using a green belt occlusal-reduction diamond bur (Frank Dental GmbH D.828.017.G.FGA, Gmund, Germany). In Group 1 and Group 2, a green belt wheel diamond bur (Meisinger 909G-031-FG Coarse 5/Pk, Neuss, Germany) is used to create a circumferential butt joint margin with a width of 2 mm. Then, a red belt conical diamond bur (Frank Dental GmbH D.845KR.016.G.FGA, Gmund, Germany) with an internal taper of 8° of the axial walls is used to prepare the pulp chamber [24]. A red belt medium round-end tapered diamond bur (Frank Dental GmbH D.850.016.FG, Gmund, Germany) was also used to round down the internal line angle, remove irregularities, and produce a flat polished surface. In Group 3 and Group 4, the entire preparation procedure was performed with the same burs used in Group 1 and Group 2. The only difference was that, unlike the first two groups, after occlusal reduction with an occlusal-reduction diamond bur and pulp chamber preparation with a conical diamond bur, a red belt modified shoulder fine W diamond bur (Meisinger 848WF-018-FG, Gmund, Germany) was used in 1 mm shoulder margin preparation. After the preparations, occlusal reductions, margin widths, and pulp chamber depths were measured by a periodontal probe and verified by a digital calliper (Digimatic, Mitutoyo Corp., Japan).

For each group, prepared teeth were scanned using a digital intraoral scanner (CEREC AC, Primescan, Dentsply Sirona, York, PA, USA) and the external CAD data was processed by Sirona InEos X5 software (InEos X5, Dentsply-Sirona, York, PA). Thus, standard tessellation language (STL) files were acquired and imported into Shapr 3D (Shapr 3D, Budapest, Hungary), a CAD software for designing and manufacturing ready models. The master dies with sets of four were designed by drawing bases under the prepared tooth STLs for different groups. Then, using a 3D printer (Asiga Ultra (50), ASIGA, Sydney, Australia) and 3D-printed model resin (VarseoWax Model, Bego, Bremer, Germany), the prepared molars were replicated into dies with a layer thickness of 50 μm. After the dies were printed, they were washed with 99% isopropanol alcohol for 3 min (Form Wash, Formlabs^®^, Somerville, USA) and post-cured twice for 20 min at 60 °C (Form Cure, Formlabs^®^, Somerville, USA) according to the manufacturer’s recommendations.

For the endocrown design, typodont maxillary molar teeth were scanned before and after preparation. These STL data were processed in exocad DentalCAD software (exocad GmbH). Endocrown designs were made on the prepared tooth STLs to reflect the initial form of the tooth. The cement space was set as 80 μm during the chairside CAD design. Then, using the 3D printer and 3D-printed ceramic-filled hybrid material (VarseoSmile Triniq, Bego, Bremer, Germany), the endocrowns were fabricated with a layer thickness of 50 μm. The 3D-printed endocrowns were washed with 99% isopropanol alcohol for 5 min (Form Wash, Formlabs^®^, Somerville, USA) and post-cured twice for 20 min at 60 °C (Form Cure, Formlabs^®^, Somerville, USA) according to the manufacturer recommendations.

The method for evaluating fit applied in this study was a 3D analysis technique using a proprietary software program (OraCheck, Cyfex AG, Zurich, Switzerland). The endocrown preparation dies were first scanned using the digital intraoral scanner and saved as the master digital file of the preparation. The inner surface of each endocrown was lightly wiped with a lubricant (Vaseline) before injecting a thin layer of light-body impression material (Elite HD + light-body; Zhermack SpA, Badia Polesine (RO), Italy). Each endocrown was loaded and seated for 4 min to obtain the complete setting time under a constant axial force of 50 N [25]. A 5 kg weight was used to standardize the applied force, corresponding to 50 N. Excess impression material was carefully removed from the margins under the dental microscope using a surgical blade (no. 12; Feather Safety Razor Co., Ltd., Osaka, Japan). As the 3D analysis method relies on the superimposition of pre- and post-seating surface scans, no luting procedure was performed to maintain the precision of digital gap measurements.

**Fig. 1:**
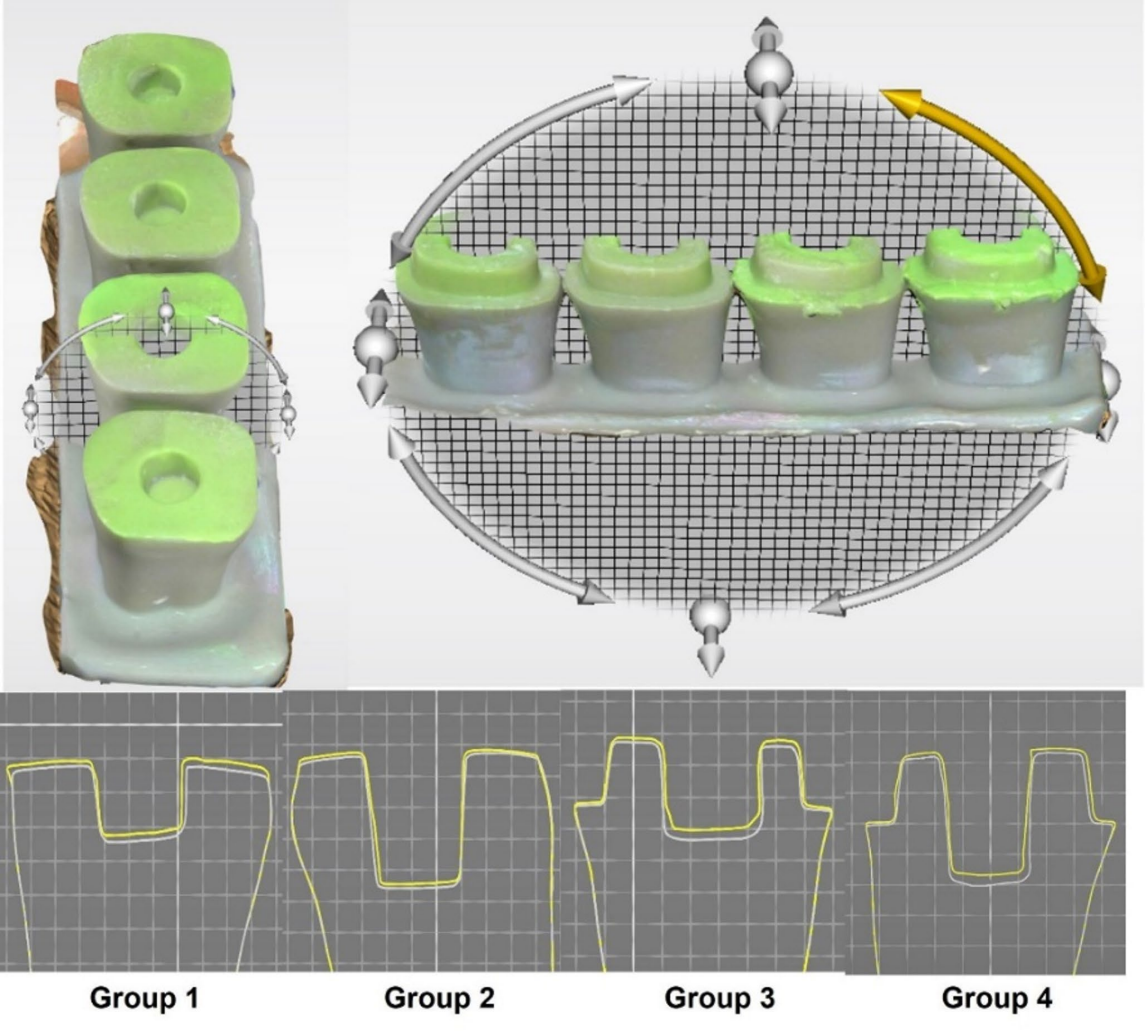
The buccolingual (BL) and mesiodistal (MD) sections chosen in the OraCheck 3D analysis software from the core region of each superimposition and the cross-sectional images of different groups on which measurements were performed

After removing the endocrown from the preparation, a second scan with the impression material covering the preparation die was performed using the digital intraoral scanner. The 3D analysis software program digitally superimposed the two recorded scans in STL files for each tested group. The subtractive analysis was accomplished by calculating the distances from each surface point of the first data set to the surface points of the second data set. The software’s best-fit algorithm selected approximately 20,000 points per surface matching [26]. The vertical sections were selected from the core region of each superimposition in the buccolingual (BL) and mesiodistal (MD) directions to evaluate the means of the marginal gap (MG), internal gap (IG), pulp chamber gap (PCG), and overall gaps (OG) of groups within all three dimensions over the superimposition views (Fig. 1).

**Fig. 2:**
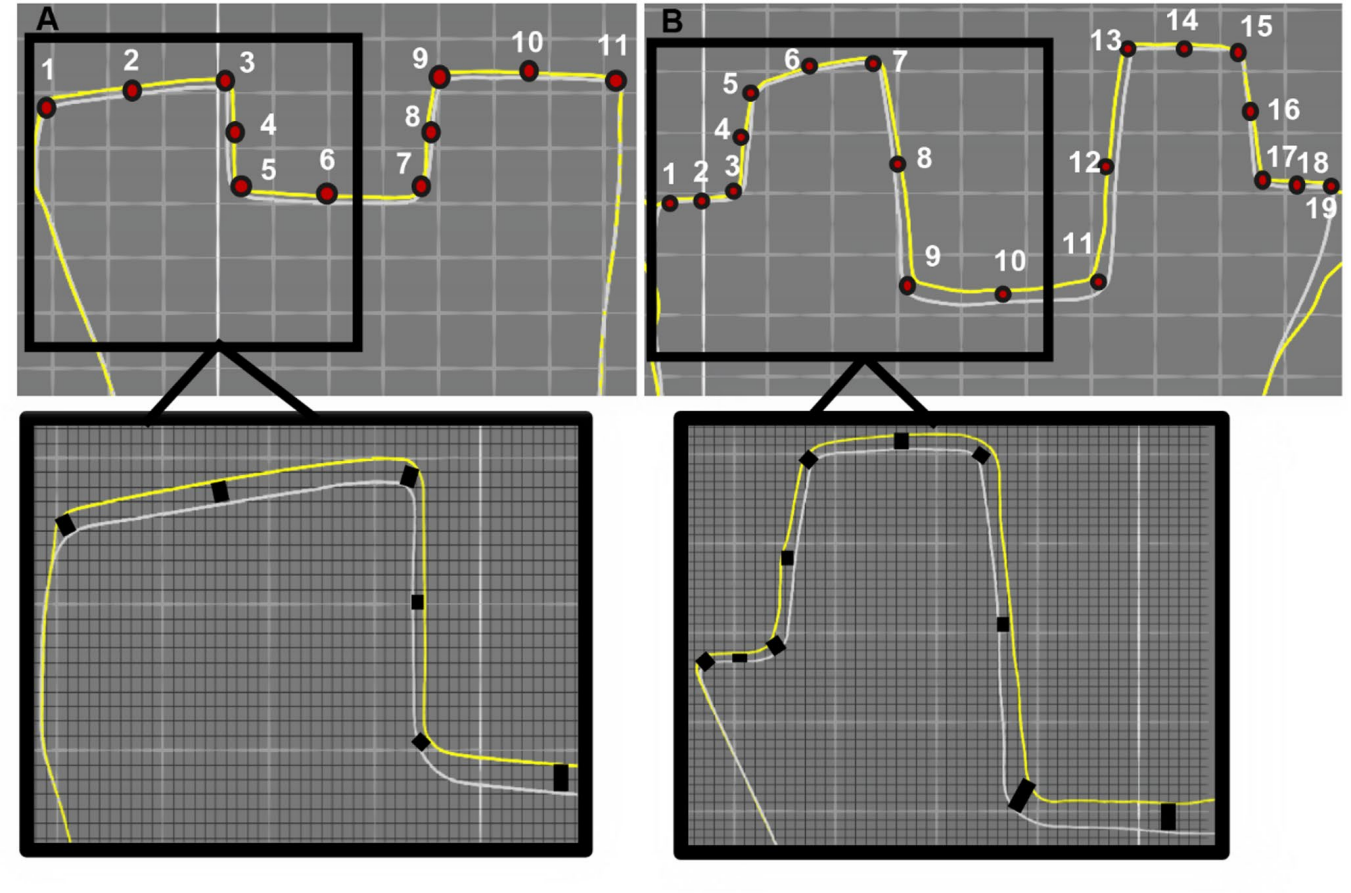
(**A**) Marginal and internal fit analysis of Group 1 from 11 points in the image taken via STL data superimposition in OraCheck 3D analysis software and gap measurement performed at relevant points in the close-up image below. (**B**) Marginal and internal fit analysis of Group 4 from 19 points in the image taken via STL data superimposition in Oracheck 3D analysis software and gap measurement performed at relevant points in the close-up image below *Images represent mesiodistal (MD) sections. *Each square is 1 mm^2^

For Group 1 and Group 2, regardless of pulp chamber depth, the mean values of points 1 and 11 in MG measurement, points 2 to 10 in IG measurement, points 3 to 9 in PCG measurement, and points 1 to 11 in OG measurement were calculated (Fig. 2A). For Group 3 and Group 4, regardless of pulp chamber depth, the means of points 1 and 19 in MG measurement, points 2 to 18 in IG measurement, points 7 to 13 in PCG measurement, and points 1 to 19 in OG measurement were calculated (Fig. 2B). The points in the pulp chamber gap assessment are included in the internal gap assessment. The overall gap assessment includes both marginal and internal gap assessment areas. Close-up cross-sectional images of the measurement points are also as in Fig. 2.

The data were analyzed using IBM SPSS V23 (SPSS; IBM Statistics, Armonk, NY) and the WRS2 package in the R program. The normality of distribution was examined with the Shapiro-Wilk Test. Pearson’s Correlation Coefficient was used to analyze the relationship between measurements that followed a normal distribution, and Spearman’s rho Correlation Coefficient was used for measurements that did not follow a normal distribution. A Two-Way Robust ANOVA was employed to compare measurements that did not follow a normal distribution according to the preparation type and preparation depth. The analysis results were presented as median (minimum-maximum). The significance level was set at *p* < 0.05.

## Results

**Fig. 3:**
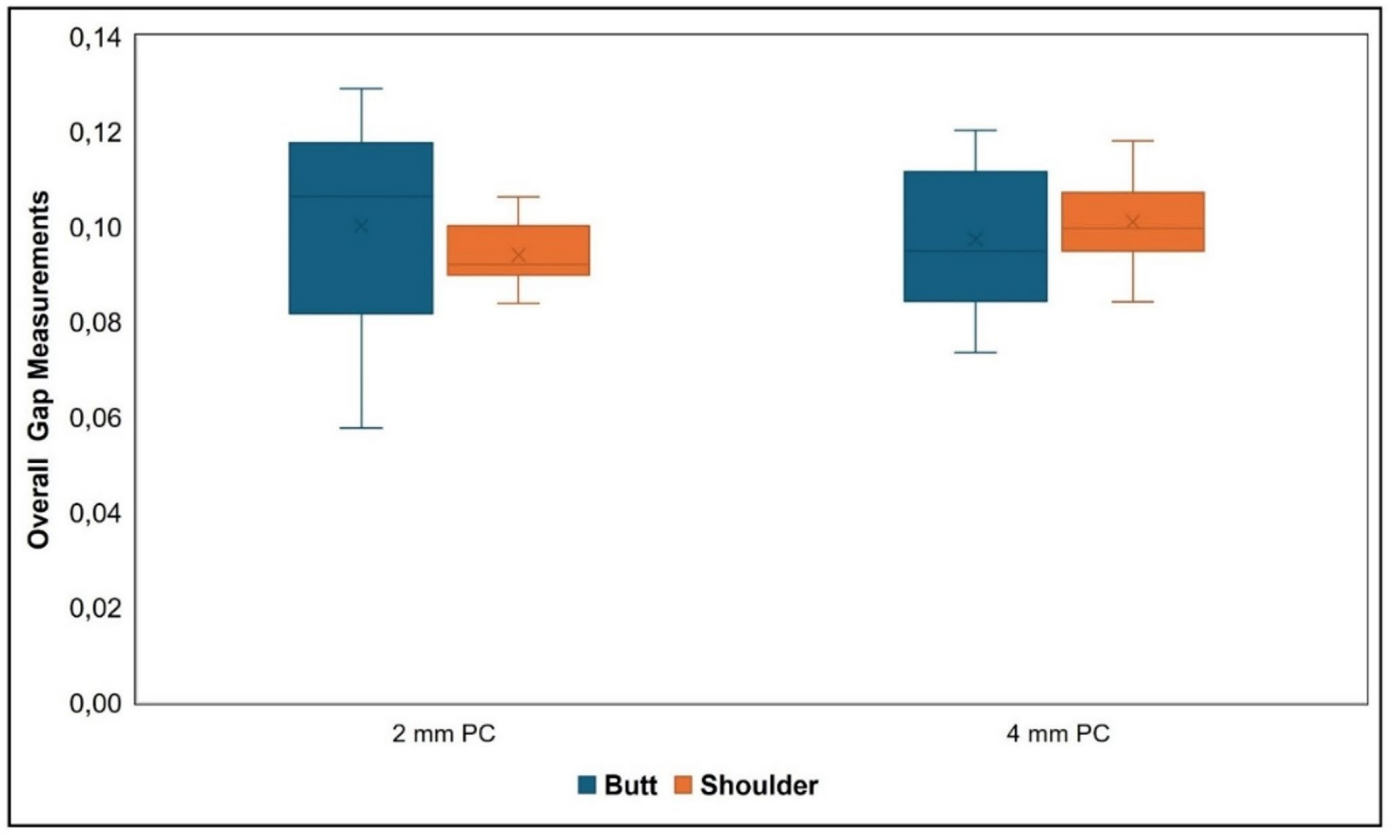
The box plot of overall gap (OG) measurements of different preparation groups

Based on the data, internal gap, marginal gap, pulp chamber gap and overall gap measurements were compared according to different preparation types and pulp chamber depths. Accordingly, the obtained values are presented in Table 1; Fig. 3. No significant differences were found between groups in terms of internal and overall gap values (*p* > 0.05), whereas statistically significant differences were observed in marginal and pulp chamber gap values (*p* < 0.05).

**Table 1:** Median (min–max) values of marginal gap, internal gap, and pulp chamber gap (mm) for different preparation groups

Marginal Gap Measurements				***p*** **value**
Pulp Chamber Depth	Preparation Type		Median	
	**Butt**	**Shoulder**		
2 mm	0.08 (0.03–0.11)^A^	0.05 (0.03–0.07)^B^	0.06 (0.03–0.11)	***p*** **= 0.024**
4 mm	0.05 (0.04–0.08)^B^	0.05 (0.02–0.08)^B^	0.05 (0.02–0.08)	
Median	0.06 (0.03–0.11)	0.05 (0.02–0.08)	0.05 (0.02–0.11)	

**p value**	***p*** **= 0.024**			
**Internal Gap Measurements**				
2 mm	0.11 (0.06–0.14)	0.10 (0.09–0.11)	0.10 (0.06–0.14)	***p*** **= 0.735**
4 mm	0.09 (0.05–0.15)	0,11 (0.09–0.12)	0.10 (0.05–0.15)	
Median	0.10 (0.05–0.15)	0.10 (0.09–0.12)	0.10 (0.05–0.15)	

***p*** **value**	***p*** **= 0.310**			
**Pulp Chamber Gap Measurements**				
2 mm	0.11 (0.07–0.14)^A^	0.10 (0.10–0.12)^A^	0.10 (0.07–0.14)	***p*** **< 0.001**
4 mm	0.09 (0.06–0.15)^A^	0.15 (0,13–0.17)^B^	0.14 (0.06–0.17)	
Median	0.10 (0.06–0.15)	0.13 (0.10–0.17)	0.11 (0.06–0.17)	
***p*** **value**	***p*** **= 0.031**			

There is no difference between interactions with the same letter.

When comparing preparation types in terms of marginal gap values, the butt preparation type had a significantly higher marginal gap value compared to the shoulder preparation type (*p* = 0.024). Comparison by pulp chamber depth showed that the marginal gap values were higher at 2 mm than at 4 mm (*p* = 0.024). The highest marginal gap was measured in Group 1 (0.08 mm), while Groups 2, 3, and 4 all exhibited lower median marginal gaps of 0.05 mm. These differences were statistically significant (*p* = 0.017) (Table 1).

Statistically significant differences were detected when comparing pulp chamber gap values (*p* < 0.05). The largest gap value was identified in the shoulder preparation type (*p* = 0.031), whereas among the pulp chamber depth types, it was recorded at 4 mm (*p* < 0.001). Group 4 showed the highest pulp chamber gap value (0.15 mm) (*p* < 0.001) (Table 1).

**Fig. 4:**
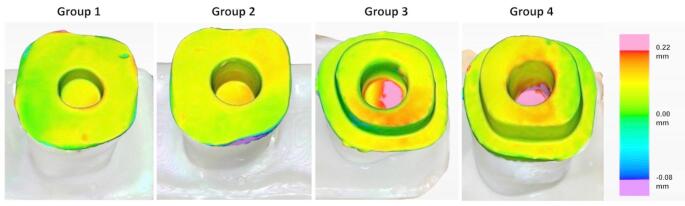
Color map of cement gap area for each group on the superimposition images obtained from Oracheck 3D analysis software *Cement gap range varies between − 0.08 and 0.22 mm

The color map of the cement gap area measurement for each group on the superimpositions was obtained from 3D analysis software, as shown in Fig. 4.

These color-coded maps visualize the cement gap distribution, with green indicating optimal adaptation (0.00 mm), red/pink representing larger gaps (up to + 0.22 mm), and blue/purple indicating tighter or negative deviations (down to − 0.08 mm). Warmer colors reflect increased gap thickness. Group 4 demonstrates the most extensive red/pink areas, particularly in the pulp chamber, indicating poor internal adaptation. This visual pattern aligns with the cement gap volume data presented in Table 2, where Group 4 exhibited the highest cement gap volume (16.62 mm³) and Group 1 (8.69 mm³) showed the lowest. These results collectively suggest that deeper pulp chambers and shoulder configurations may compromise internal adaptation in 3D-printed endocrowns.

**Table 2:** The cement gap volume values (mm3) of different Preparation groups

Cement Gap Volume (mm^3^) Measurements		
Pulp Chamber Depth	Preparation Type	
	Butt	Shoulder
2 mm	8.69	12.85
4 mm	10.21	16.62

## Discussion

The adaptation of endocrown restorations within acceptable marginal and internal fit is critical for long-term clinical outcomes [27]. Developing 3D printing technologies is expected to improve the adaptation of endocrown restorations, which is greatly affected by the tooth preparation design, and to affect their longevity [4, 16, 28, 29]. This study evaluated the influence of different preparation designs on the marginal and internal fit of 3D-printed permanent endocrowns. The results revealed significantly higher marginal gap values in Group 1 and greater pulp chamber gaps in Group 4. However, no significant differences were found between preparation designs in terms of internal fit. Therefore, the first null hypothesis was rejected, while the second was accepted.

The margin design of endocrowns can significantly impact their mechanical performance, as well as both marginal and internal fit [7–10]. Farghal et al. [13] reported that butt joint preparations provided superior marginal and internal fit fcompared to 1- and 2-mm ferrule (shoulder) preparations in CAD/CAM-fabricated endocrowns, irrespective of the ceramic material used. However, their study also indicated that shoulder preparations offered greater retention. Similarly, Seo et al. [14] found that a 1 mm shoulder finish line enhances bonding potential by increasing the number of axial walls from two (in butt joint) to four, thereby expanding the bonding surface area. They also noted that this increased surface area may improve marginal adaptation. In the present study, a shoulder finish line demonstrated better marginal fit than the butt joint, possibly due to its greater axial wall height, which may have enhanced mechanical retention and facilitated more complete seating of the restoration. This aligns with the findings of the aforementioned studies. Additionally, unlike the prior investigations that used conventional CAD/CAM methods, the use of 3D printing in this study may have further contributed to improved marginal adaptation. Supporting this, a previous study comparing CAD/CAM hybrid materials with 3D-printed ceramic-filled hybrid crowns found that the 3D-printed group exhibited superior marginal adaptation, likely due to its lower flexural modulus and the inherent precision of the additive manufacturing process rather than material composition alone [18].

The depth of the pulp chamber in endocrown preparations plays a crucial role, as a deeper pulpal cavity is assumed to provide more surface area for adhesive bonding and facilitate better transmission of masticatory forces [30]. Several studies have indicated that increasing the cavity depth may also lead to greater marginal discrepancies [31, 32]. For example, Shin et al. [31] found that endocrowns with a 4-mm intraradicular extension exhibited significantly larger marginal and internal gaps than those with a 2-mm depth. However, contrary to these findings, the present study revealed that a 2 mm pulp chamber depth resulted in significantly higher marginal gap values compared to the 4 mm depth. This discrepancy may be explained by the potential for greater restoration displacement during seating in shallower cavities, whereas increased depth may offer a mechanical locking effect that enhances seating accuracy. In terms of internal fit, the present results are in agreement with the study by Darwish et al. [33], which reported no significant differences between endocrowns prepared with 3 mm and 5 mm pulp chamber depths. Similarly, no significant differences in internal fit were observed in this study between the 2 mm and 4 mm depth groups. This consistency may be attributed to the high fabrication precision achieved by using 3D printing technology with a fine recommended layer thickness of 50 μm.

The pulp chamber has frequently been identified as the area with the poorest fit in endocrown restorations [34–36]. In light of this, the present study specifically evaluated the pulp chamber gap to gain a more detailed understanding of the internal adaptation. The results revealed higher pulp chamber gap values in the 4 mm depth groups compared to those with 2 mm depth. This discrepancy may be attributed to the narrow and complex anatomy of the pulp chamber, which can pose challenges for accurate digital impression capture and result in blurred imaging in deeper regions [34–37]. Additionally, the flow characteristics of the light-body impression material used in this study might have been hindered in deeper cavities, where limited escape pathways could lead to material entrapment and subsequently increased measured gap values.

In this study, unlike previous investigations, the cement gap volume was also measured to provide a more comprehensive evaluation of overall adaptation. The highest cement gap volume was observed in Group 4, which combined a shoulder preparation with a 4 mm pulp chamber depth. According to Taha et al. [38], a 1 mm ferrule (shoulder) preparation design may help reduce shear stresses on axial walls and improve load distribution along the margins. Additionally, Farghal et al. [13] noted that reduced axial wall dimensions in butt-joint designs may result in thinner cement layers compared to bulkier shoulder designs. However, based on the findings of the present study, the combination of a deeper pulp chamber and taller axial walls in the shoulder configuration may have impeded the escape of the fit-indicating material, causing material entrapment and increased cement gap volume. Similarly, Shin et al. [31] reported that endocrowns with 4 mm pulp chamber depths exhibit greater volume and surface area, contributing to increased marginal and internal discrepancies. Consistent with their findings, our study found that Group 4 showed both the greatest cement gap volume and the highest pulp chamber gap values, reinforcing the notion of compromised internal fit. Although 2 mm pulp chamber groups had larger marginal gaps, they exhibited lower cement gap volumes, suggesting that depth may have a more pronounced effect on internal discrepancy.

To strengthen the validity of this study, a preliminary pilot investigation was conducted, and the methodology was refined accordingly. Typodont teeth were selected to ensure standardization and high clinical relevance, despite not using natural teeth. All preparations were performed by a single operator to minimize procedural variability. Given the ceramic-filled hybrid microstructure of the 3D-printed endocrown material, a cement gap of 80 μm was designated during the CAD design phase, balancing the commonly recommended settings of 60 μm for ceramics and 120 μm for resin composites [27]. Although two-dimensional (2D) techniques are frequently used to evaluate marginal and internal fit, this study employed a 3D digital analysis method to achieve more precise measurements without data loss, enabling multipoint measurements across the entire internal surface and from different cross-sections.

This in vitro study has some limitations. A single type of digital intraoral scanner, 3D printer, and 3D-printed material was used in this study. Contributions to the literature can be made with future studies employing different materials and technologies. Additionally, the restorations were not cemented in the present study to preserve the precision of 3D digital fit measurements, which may have influenced marginal gap values. Future investigations should include definitive cementation protocols to evaluate their impact on adaptation. Furthermore, the influence of thermomechanical aging or fatigue loading on the fit and long-term performance of 3D-printed endocrowns remains unexplored. These factors should be addressed in future in vitro and in vivo studies, as they may significantly affect the clinical durability and behavior of 3D-printed restorations.

## Conclusions

A butt joint design with a 2 mm pulp chamber depth led to poor marginal fit, while deeper pulp chambers in shoulder designs compromised internal adaptation. Therefore, to achieve optimal marginal and internal fit in clinical practice, a 1 mm shoulder preparation with a 2 mm pulp chamber depth is recommended for 3D-printed permanent endocrown restorations.

## Data Availability

No datasets were generated or analysed during the current study.
